# Altered Magnesium Environments Restrict Colorectal HT-29 Spheroid Growth by Disturbing Cellular Mg^2+^ Homeostasis

**DOI:** 10.3390/ijms27020834

**Published:** 2026-01-14

**Authors:** Nattida Kampuang, Pongsakorn Lapchock, Tanida Treerattanakulporn, Phossawee Kongkaew, Siriporn Chamniansawat, Narongrit Thongon

**Affiliations:** Department of Medical Sciences, Faculty of Allied Health Sciences, Burapha University, Chonburi 20131, Thailandpongsakornlapchock@gmail.com (P.L.); tanidatrirattanakulporn@gmail.com (T.T.); garfield29phos@gmail.com (P.K.); siripornc@buu.ac.th (S.C.)

**Keywords:** colorectal tumor spheroids, magnesium imbalance, low extracellular Mg^2+^ environment, high extracellular Mg^2+^ environment, Mg^2+^ transport mechanisms, TRPM6/7 channel regulation, tumor microenvironment

## Abstract

Dysregulated magnesium (Mg^2+^) homeostasis contributes to colorectal cancer (CRC), yet its context-dependent function within the tumor microenvironment remains unresolved. This study aimed to determine how sustained low and high extracellular Mg^2+^ environments affect CRC spheroid (SP) growth and Mg^2+^ homeostasis using HT-29 SPs. We analyzed Mg^2+^ flux, the expression of Mg^2+^ transporters (e.g., Transient Receptor Potential Melastatin (TRPM) 6), viability, apoptotic and autophagic markers, and phospho-/oxidoproteomic alterations. Both Mg^2+^ extremes destabilized SP architecture, reduced viability, and induced apoptosis and autophagy, with SPs displaying heightened vulnerability relative to 2D cultures. Mg^2+^ stress impaired Mg^2+^ influx and eliminated adaptive transporter regulation in SPs. Loss of membrane TRPM6/7 heterodimers, driven by altered phosphorylation (e.g., TRPM6 Serine 141, Serine 1252, Threonine 1851) and elevated oxidation (e.g., Methionine 1755), suppressed channel activity. High Mg^2+^ caused profound metabolic failure despite increased total Mg^2+^, reflecting functional Mg^2+^ deficiency. CRC spheroids are acutely susceptible to Mg^2+^ imbalance due to collapsed transporter homeostasis and post-translational inhibition of Mg^2+^ channels. These findings reveal a targetable metabolic vulnerability and support the therapeutic potential of localized Mg^2+^ modulation in CRC.

## 1. Introduction

Dysregulated magnesium (Mg^2+^) homeostasis is increasingly linked to colorectal cancer (CRC) initiation and progression [1,2], yet its mechanistic contribution remains unresolved and highly context-dependent. Epidemiological evidence reflects this complexity: while an insufficient dietary magnesium intake elevates the risk of CRC [3,4], other studies paradoxically associate excessive intake with a greater incidence [5]. At the tissue level, CRC specimens frequently exhibit heightened intracellular Mg^2+^ compared with adjacent normal mucosa [6,7], likely driven by increased uptake through Transient Receptor Potential Melastatin (TRPM)6, TRPM7, and Magnesium Transporter 1 (MagT1), coupled with reduced efflux via Cyclin M4 (CNNM4) [8,9,10,11]. Elevated intracellular Mg^2+^ correlates with enhanced doxorubicin (DXR) resistance, cancer stemness, spheroid (SP) stability, and invasive behavior in CRC cells [12,13]. However, in contrast to tumor profiles, experimental exposure to supraphysiologic Mg^2+^ (15–30 mM) induces cell-cycle arrest and apoptosis in CRC cells [14]. Despite suggesting therapeutic utility, such concentrations (>5 mM) constitute severe clinical hypermagnesemia in vivo, causing marked cardiovascular and neurological toxicity [15]. This discrepancy between the anti-tumor effects of extreme Mg^2+^ in vitro and their systemic hazards in vivo highlights the dualistic and poorly defined nature of Mg^2+^ in tumor biology. It underscores the need to identify clinically relevant thresholds.

Our previous work clarified this paradox by showing that HT-29 cells grown as SPs exhibit higher membrane TRPM6 and TRPM6/7 expression, increased Mg^2+^ influx, elevated intracellular Mg^2+^, and greater stemness and migratory capacity than their parental adherent counterparts under control (1.0 mM Mg^2+^) conditions [13]. Notably, pharmacological inhibition of TRPM6 and TRPM6/7 sharply reduced SP Mg^2+^ influx, intracellular Mg^2+^, cancer stemness, SP stability, and migration [13]. However, a central and unresolved question persists: how do sustained low and high Mg^2+^ environments directly modulate cellular Mg^2+^ homeostasis and CRC SP progression? Our previous study showed that pharmacological inhibition disrupts SP growth, but the effects of physiological extremes of extracellular Mg^2+^ and their underlying molecular mechanisms remain unexplored.

The tumor microenvironment (TME) comprises a complex network of cellular and non-cellular components that collectively drive tumor progression by supporting unchecked proliferation, evasion of apoptosis, and metastasis. Accordingly, targeting the TME has become a promising strategy for treating solid tumors [16]. Modulating Mg^2+^ within the TME has recently gained attention. Localized Mg^2+^-release systems using microsphere-encapsulated hydrogels can remodel the immune microenvironment and enhance the efficacy of immune checkpoint blockade in CRC [17]. Likewise, Mg-based biomaterials show potential for improving tumor treatment [18], highlighting the therapeutic value of precisely controlling tumoral Mg^2+^ fluxes. However, the direct mechanistic effects of Mg^2+^ on core cancer-cell hallmarks within the solid TME remain largely undefined.

To address this gap, we examined how extracellular Mg^2+^ availability shapes CRC SP progression and Mg^2+^ homeostasis. We used HT-29 SPs as a physiologically relevant 3D model that recapitulates the structural and microenvironmental features of solid tumors [19]. HT-29 cells, derived from colorectal adenocarcinoma (ATCC HTB-38), form robust SPs that mimic key aspects of CRC biology, including cell–cell interactions, nutrient gradients, and drug resistance mechanisms, providing a robust platform for probing ion dysregulation. We hypothesized that both low and high Mg^2+^ would disrupt Mg^2+^ homeostasis in CRC SPs, leading to structural disintegration and cell death through dysregulated TRPM6/7 signaling and mitochondrial dysfunction. Our objective was to determine how sustained deviations from physiological Mg^2+^ levels affect SP integrity, cell survival, Mg^2+^ transporter function, cellular Mg^2+^ homeostasis, and post-translational regulation of TRPM6/7 channels. We analyzed major Mg^2+^ transporters, including TRPM6, TRPM7, MagT1, CNNM4, and mitochondrial inner-membrane magnesium transporter 2 (Mrs2). Confocal and 4D microscopy enabled visualization of Mg^2+^ influx and channel localization, respectively, which we integrated with high-resolution global, phospho, and oxidoproteomic profiling to identify underlying signaling and stress pathways. This multi-modal strategy was designed to define how Mg^2+^ dysregulation affects CRC SPs and to evaluate its therapeutic relevance.

## 2. Results

### 2.1. Low and High Mg^2+^ Conditions Disrupted HT-29 SPs

**Figure 1:**
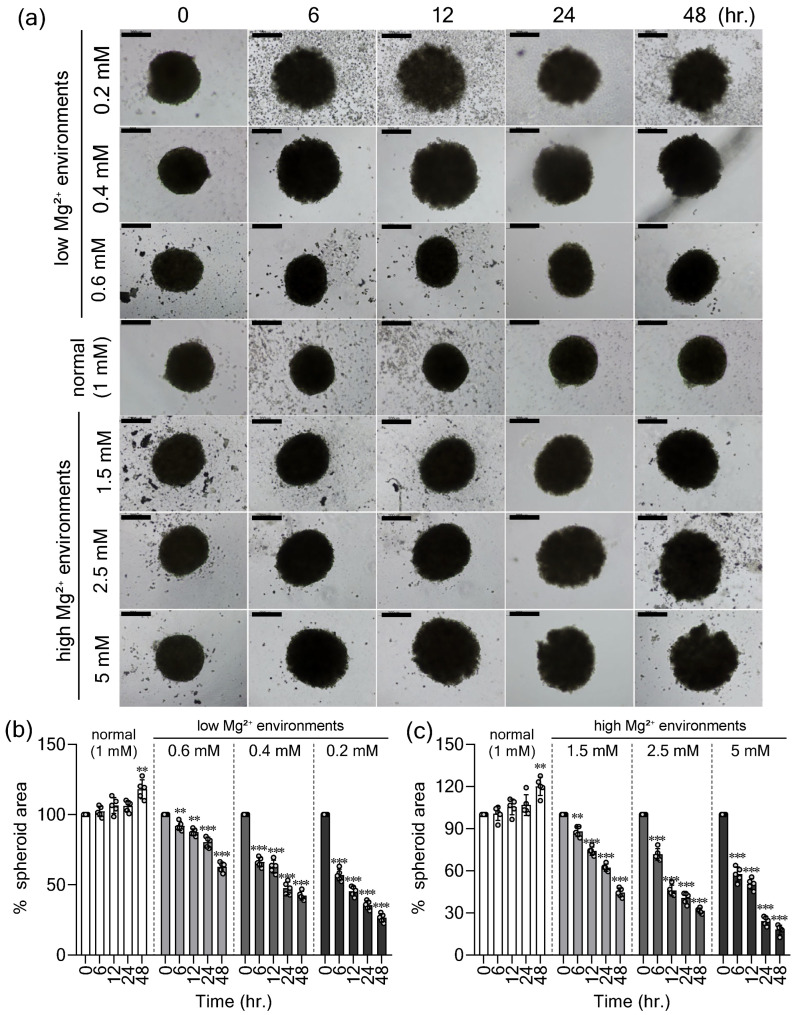

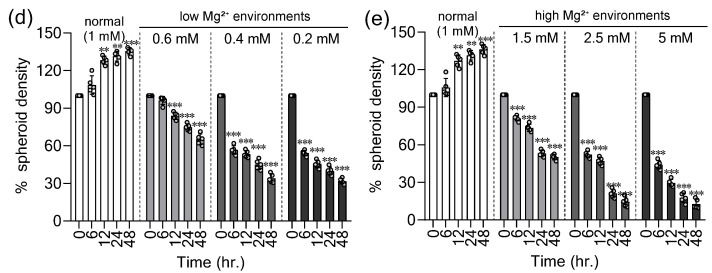
Deviations from physiological extracellular Mg^2+^ disrupt HT-29 spheroid integrity. (**a**) Representative bright-field images of spheroids (SPs) after 48 h in control (1.0 mM Mg^2+^), low Mg^2+^ (0.6, 0.4, 0.2 mM), and high Mg^2+^ (1.5, 2.5, 5.0 mM) media, reflecting mild, moderate, and severe deviations, respectively. Scale bar: 200 µm. We conducted quantitative analyses of the SP area under low Mg^2+^ conditions (**b**) and high Mg^2+^ conditions (**c**), as well as the SP structural-integrity scores under low Mg^2+^ conditions (**d**) and high Mg^2+^ conditions (**e**). Data are mean ± SD (n = 5). Statistical analysis was performed using one-way ANOVA with Dunnett’s post hoc test. The *p*-values are as follows: ** *p* < 0.01, and *** *p* < 0.001 versus the corresponding 0 h group.

To determine how extracellular Mg^2+^ affects SP stability, HT-29 SPs were generated and then maintained in control (1.0 mM Mg^2+^), low Mg^2+^ (0.6, 0.4, 0.2 mM), or high Mg^2+^ (1.5, 2.5, 5 mM) media, representing mild to severe deviations from physiological levels [15]. Under physiological conditions, SPs retained a compact spherical morphology with a dense core and defined borders (Figure 1a), and their area (Figure 1b) and structural integrity (Figure 1d) increased steadily over 48 h, confirming the robust growth of our CRC SP model. In contrast, both low and high Mg^2+^ induced rapid structural disruption (Figure 1a). By 24 h, peripheral cell shedding indicated reduced intercellular cohesion, and by 48 h, moderate and severe Mg^2+^ deviations caused extensive disaggregation and loss of spherical architecture (Figure 1a). Quantitative analyses demonstrated that both conditions significantly and dose-dependently reduced SP area (Figure 1b,c) and integrity scores (Figure 1d,e) in comparison to control conditions. These findings indicate that deviations from physiological extracellular Mg^2+^ in either direction compromise the structural integrity and growth of CRC SPs.

### 2.2. Low and High Mg^2+^ Conditions Induced Cell Death in HT-29 SPs

**Figure 2:**
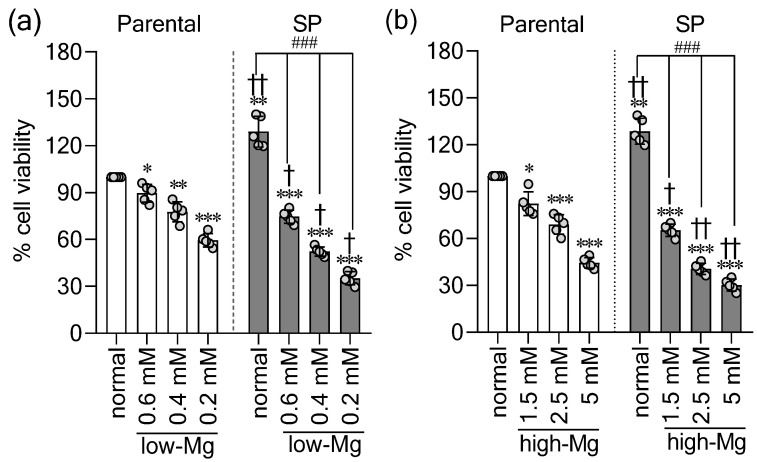
Spheroids show increased sensitivity to extracellular Mg^2+^ imbalance. Cell viability was measured by WST-1 assay in parental (2D) and spheroid (SP)-derived (3D) HT-29 cells after 48 h in (**a**) low Mg^2+^ media (low-Mg) and (**b**) high Mg^2+^ media (high-Mg). Data are mean ± SD (n = 5). Statistical analysis was performed using one-way ANOVA with Dunnett’s post hoc test for multiple comparisons versus control groups and unpaired Student’s *t*-test for comparisons between parental and SP groups under the same Mg^2+^ condition. The significance levels are as follows: * *p* < 0.05, ** *p* < 0.01, *** *p* < 0.001 versus the normal parental group. † *p* < 0.05, †† *p* < 0.01 versus parental cells under the same Mg^2+^ condition. ### *p* < 0.001 versus the control SP group.

Structural disintegration of SPs under non-physiological Mg^2+^ was accompanied by peri-spheroidal vesicles, consistent with a cellular stress response. We first assessed the baseline metabolic activity and found that SP-derived cells exhibited significantly higher activity—a proxy for viability—than adherent parental HT-29 cells at comparable passage (Figure 2a,b), confirming their elevated proliferative and metabolic state. We subsequently investigated the functional consequence of Mg^2+^ dysregulation. Both low and high Mg^2+^ conditions markedly reduced the viability of parental and SP-derived cells in a dose-dependent manner (Figure 2a,b). Although SPs originate from a more robust population, their viability under Mg^2+^ stress fell below that of the corresponding treated parental groups, indicating that the SP phenotype, despite its resilience under physiological conditions, is more vulnerable to deviations in extracellular Mg^2+^.

**Figure 3:**
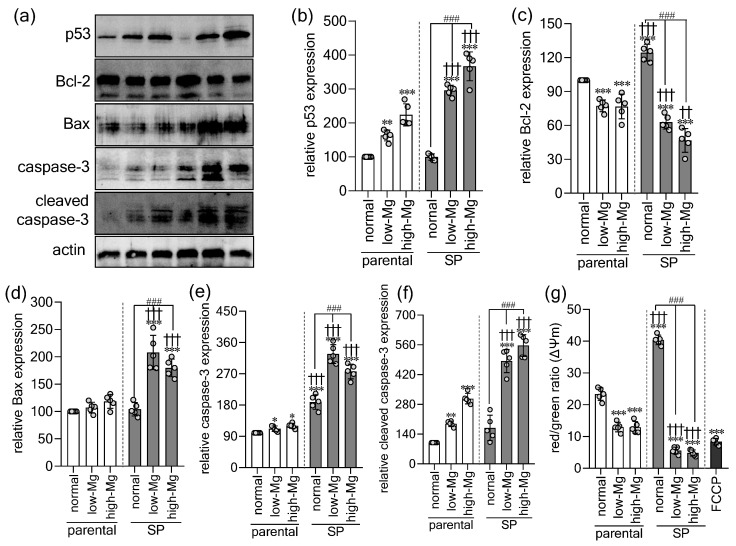
Spheroids show heightened apoptotic sensitivity to extracellular Mg^2+^ imbalance. (**a**) Representative Western blots of p53, Bcl-2, Bax, caspase-3, and cleaved caspase-3 in parental (2D) and spheroid-derived (SP; 3D) HT-29 cells cultured under control, moderate low Mg^2+^ (0.4 mM; low-Mg), and moderate high Mg^2+^ (2.5 mM; high-Mg) conditions. We conducted a quantitative analysis of (**b**) p53, (**c**) Bcl-2, (**d**) Bax, (**e**) caspase-3, and (**f**) cleaved caspase-3. (**g**) Mitochondrial membrane potential (ΔΨm) was measured by the red-to-green JC-1 fluorescence intensity ratio; FCCP served as a depolarization control. Data are mean ± SD (n = 5). Statistical analysis was performed using one-way ANOVA with Dunnett’s post hoc test for multiple comparisons versus control groups and unpaired Student’s *t*-test for comparisons between parental and SP groups under the same Mg^2+^ condition. The significance levels are as follows: * *p* < 0.05, ** *p* < 0.01, *** *p* < 0.001 versus the control parental group. †† *p* < 0.01, ††† *p* < 0.01 versus parental cells under identical Mg^2+^ conditions. ### *p* < 0.001 versus the control SP group.

To elucidate the mechanisms driving the observed viability loss, we examined key apoptotic regulators, including tumor suppressor protein p53 (p53), B-cell lymphoma 2 (Bcl-2), Bcl-2-associated X protein (Bax), and caspase-3 (Figure 3a) [20,21]. Both moderate low and high Mg^2+^ markedly increased p53 in parental cells (Figure 3b), whereas SP-derived cells showed a stronger induction of p53 under Mg^2+^ stress, exceeding both control SPs and stressed parental cells. This heightened responsiveness extended to Bcl-2 regulation. Under physiological conditions, SPs upregulated Bcl-2 relative to parental controls (Figure 3c), but low and high Mg^2+^ conditions significantly suppressed Bcl-2 in both models. As a result, SPs consistently exhibited lower Bcl-2 than treated parental cells, indicating an impaired anti-apoptotic response. A pro-apoptotic shift in SPs was further supported by a significant increase in Bax (Figure 3d), which remained unchanged in parental cells. Correspondingly, Mg^2+^ stress activated the apoptotic effector pathway, with total and cleaved caspase-3 significantly elevated in all treated groups (Figure 3e,f), and more strongly in SPs. Because apoptosis entails loss of mitochondrial membrane integrity, we assessed mitochondrial membrane potential (ΔΨm) using the JC-1 assay (Figure 3g). Control SPs displayed higher ΔΨm than parental cells, consistent with greater metabolic activity. However, both low and high Mg^2+^ significantly reduced ΔΨm in both models, with a more severe decline in SPs. Together, these findings demonstrate that although Mg^2+^ stress triggers apoptosis in both models, SPs exhibit a markedly heightened susceptibility, characterized by amplified p53 induction, reduced Bcl-2 expression, elevated Bax levels, enhanced caspase-3 activation, and a pronounced mitochondrial collapse.

**Figure 4:**
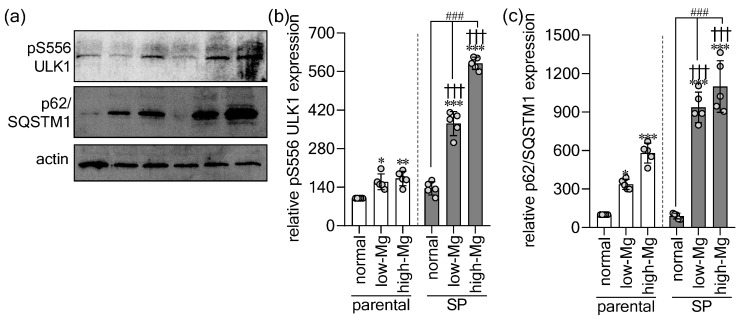
Spheroids show increased autophagic sensitivity to extracellular Mg^2+^ imbalance. (**a**) Western blots of pS556-ULK1 and p62/SQSTM1 in parental (2D) and spheroid (SP)-derived (3D) HT-29 cells exposed to control, moderate low Mg^2+^ (0.4 mM; low-Mg), and moderate high Mg^2+^ (2.5 mM; high-Mg) conditions. The quantitative analysis of (**b**) pS556-ULK1 and (**c**) p62/SQSTM1 protein expression. Data are mean ± SD (n = 5). Statistical analysis was performed using one-way ANOVA with Dunnett’s post hoc test for multiple comparisons versus control groups and unpaired Student’s *t*-test for comparisons between parental and SP groups under the same Mg^2+^ condition. The significance levels are as follows: * *p* < 0.05, ** *p* < 0.01, *** *p* < 0.001 versus the control parental group. ††† *p* < 0.001 versus parental cells under the same Mg^2+^ condition. ### *p* < 0.001 versus the control SP group.

Given that p53 can induce autophagy under cellular stress [22] and that it was markedly upregulated in Mg^2+^-stressed SPs, we next examined the autophagic response. We assessed key markers, including Unc-51-like kinase 1 phosphorylated at Serine 556 (pS556 ULK1), which initiates autophagy, and Sequestosome-1 (p62/SQSTM1), which is degraded during effective autophagic flux (Figure 4a). Both low and high Mg^2+^ conditions significantly elevated pS556 ULK1 (Figure 4b) and p62/SQSTM1 (Figure 4c) relative to their control conditions. This induction was substantially stronger in SP-derived cells than in parental cells exposed to the same Mg^2+^ stress.

Collectively, our findings show that the SP phenotype heightens sensitivity to Mg^2+^ dysregulation. This susceptibility is characterized by a dominant pro-apoptotic program, marked by reduced Bcl-2 and increased caspase activation, accompanied by a parallel, amplified autophagic response, ultimately resulting in extensive cell death.

### 2.3. Low and High Mg^2+^ Conditions Altered Mg^2+^ Homeostasis in HT-29 SPs

**Figure 5:**
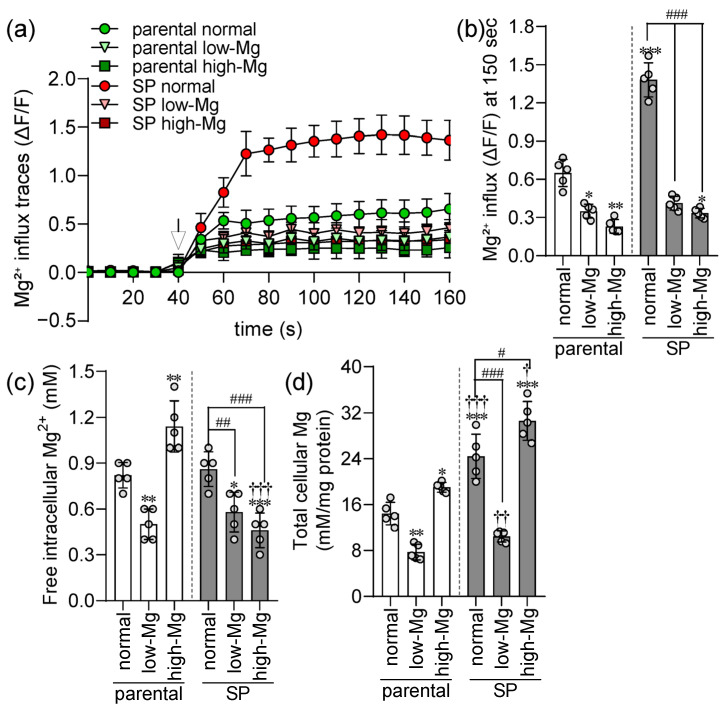
Spheroids display disrupted Mg^2+^ homeostasis under extracellular Mg^2+^ stress. (**a**) Kinetic Mg^2+^ influx traces (ΔF/F) in parental (2D) and spheroid-derived (SP, 3D) HT-29 cells following a 20 mM MgSO_4_ (indicated by arrow) under control, moderate low Mg^2+^ (0.4 mM; low-Mg), and moderate high Mg^2+^ (2.5 mM; high-Mg) conditions. (**b**) Quantitative analysis of Mg^2+^ influx rates at 150 s post-pulse. (**c**) Steady-state free intracellular Mg^2+^ levels and (**d**) total cellular Mg^2+^ content. Data are mean ± SD (n = 5). Statistical analysis was performed using one-way ANOVA with Dunnett’s post hoc test for multiple comparisons versus control groups and unpaired Student’s *t*-test for comparisons between parental and SP groups under the same Mg^2+^ condition. * *p* < 0.05, ** *p* < 0.01, *** *p* < 0.001 vs. control parental; † *p* < 0.05, †† *p* < 0.01, ††† *p* < 0.001 vs. parental under identical Mg^2+^ conditions; # *p* < 0.05, ## *p* < 0.01, ### *p* < 0.001 vs. control SP group.

To assess early disturbances in cellular Mg^2+^ regulation before overt cell death, parental and SP HT-29 cells were exposed to moderate low and high Mg^2+^ conditions for 24 h. Intracellular Mg^2+^ dynamics were tracked using ΔF/F fluorescence, with baseline acquisition during the initial 30 s, followed by a 20 mmol/L MgSO4 challenge from 40 to 160 s. After MgSO4 addition, the parental cells showed a rapid rise in Mg^2+^ that plateaued (Figure 5a). As previously reported [13], SPs displayed a significantly greater Mg^2+^ influx rate under physiological conditions (Figure 5a,b). Remarkably, Mg^2+^ stress uncovered a core defect in the SP phenotype. Whereas parental cells exhibited only a mild influx reduction, SPs showed a severe suppression of Mg^2+^ influx under both low and high Mg^2+^ conditions (Figure 5b). We then assessed steady-state free intracellular Mg^2+^ concentrations. Reflecting their impaired influx, both Mg^2+^ stresses significantly reduced free Mg^2+^ in SPs (Figure 5c). In contrast, parental cells retained regulatory responses, with low Mg^2+^ lowering and high Mg^2+^ elevating free Mg^2+^. Total cellular Mg^2+^ content showed parallel trends: low Mg^2+^ decreased, and high Mg^2+^ increased total Mg^2+^ in parental cells (Figure 5d). Control SPs contained substantially more total Mg^2+^ than parental cells, consistent with their elevated basal influx, and this baseline and this elevation were similarly modulated by extracellular Mg^2+^. Overall, these findings reveal a fundamental divergence in Mg^2+^ handling: parental cells preserve influx regulation to buffer extracellular shifts, whereas SPs, despite higher baseline capacity, lose regulatory control and experience pronounced Mg^2+^ homeostatic disruption under stress.

### 2.4. Low and High Mg^2+^ Conditions Altered Magnesiotropic Protein Expression

**Figure 6:**
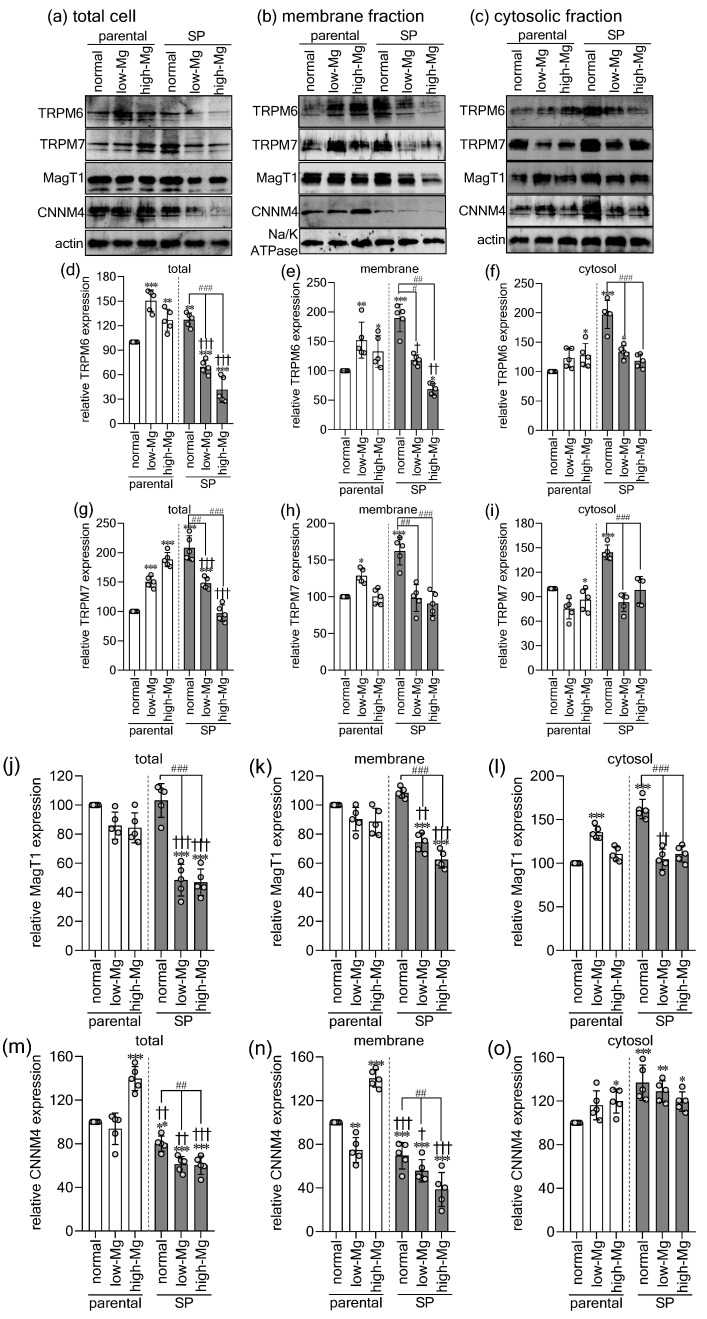
Spheroids fail to regulate Mg^2+^ transporter expression under extracellular Mg^2+^ stress adaptively. Representative Western blots of TRPM6, TRPM7, MagT1, and CNNM4 in total lysates (**a**), membrane (**b**), and cytosolic fractions of parental (2D) and spheroid-derived (3D) HT-29 cells exposed to control, moderate low Mg^2+^ (0.4 mM; low-Mg), and moderate high Mg^2+^ (2.5 mM; high-Mg) conditions. Quantification of transporter abundance in total (t), membrane (m), and cytosolic (**c**) fractions is shown for TRPM6 (**d**–**f**), TRPM7 (**g**–**i**), MagT1 (**j**–**l**), and CNNM4 (**m**–**o**). Data are mean ± SD (n = 5). Statistical analysis was performed using one-way ANOVA with Dunnett’s post hoc test for multiple comparisons versus control groups and unpaired Student’s *t*-test for comparisons between parental and SP groups under the same Mg^2+^ condition. * *p* < 0.05, ** *p* < 0.01, *** *p* < 0.001 versus the control parental group. † *p* < 0.05, †† *p* < 0.01, ††† *p* < 0.001 versus parental cells under the same Mg^2+^ condition; # *p* < 0.05, ## *p* < 0.01, ### *p* < 0.001 versus control SP group.

To elucidate the molecular basis of Mg^2+^ dysregulation in SPs, we assessed the expression and subcellular distribution of major Mg^2+^ transporters such as TRPM6, TRPM7, MagT1, and the efflux protein CNNM4 in total cell lysates, membranes, and cytosolic fractions (Figure 6a–c). Mirroring CRC tissue profiles [6,7], SPs under physiological conditions showed a marked shift toward Mg^2+^ acquisition. Relative to parental cells, SPs displayed substantial increases in total, membrane, and cytosolic TRPM6 (Figure 6d–f) and TRPM7 (Figure 6g–i). Only cytosolic MagT1 was elevated in control SPs, with no change in total or membrane pools (Figure 6j–l), indicating that enhanced membrane abundance of TRPM6/7 and likely their heterodimers primarily drives the heightened Mg^2+^ influx. Concurrently, SPs exhibited reduced total and membrane-bound CNNM4 but increased cytosolic CNNM4 (Figure 6m–o), a redistribution that aligns with their elevated Mg^2+^ influx and total Mg^2+^ content. Parental cells showed coherent, condition-specific adaptations. Under moderate low Mg^2+^, they upregulated TRPM6, TRPM7, and MagT1 while suppressing CNNM4, thereby enhancing net Mg^2+^ uptake (Figure 6d–o). Under moderate high Mg^2+^, they robustly induced total, membrane, and cytosolic CNNM4 to promote Mg^2+^ efflux (Figure 6m–o), reflecting intact epithelial homeostasis. In contrast, SPs failed to mount these selective responses. Both low and high Mg^2+^ conditions triggered downregulation of TRPM6, TRPM7, MagT1, and CNNM4 across all fractions (Figure 6d–o). This nonselective suppression of Mg^2+^ transport reveals a fundamental breakdown in regulatory control, highlighting the SP phenotype’s inability to maintain Mg^2+^ homeostasis under stress.

### 2.5. Mg^2+^ Dysregulation Disrupts TRPM6/7 Heterodimer Stability in HT-29 SPs

**Figure 7:**
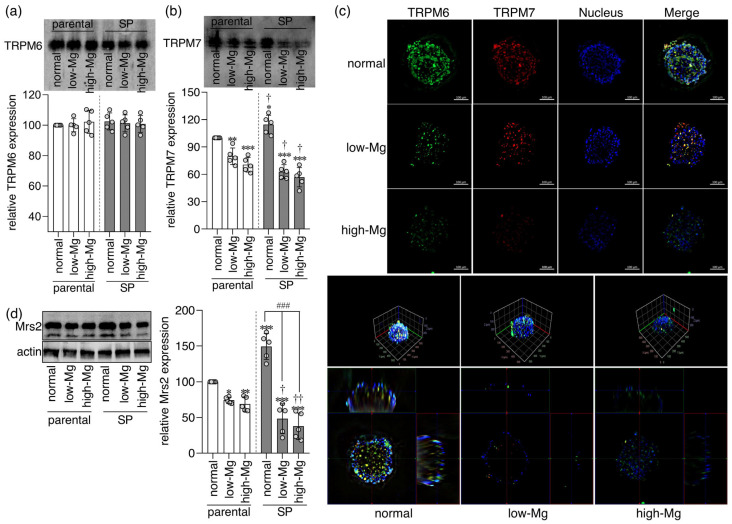
Mg^2+^ dysregulation diminishes membrane TRPM6/7 heterodimerization in spheroids. Membrane fractions from parental (2D) and spheroid (SP)-derived (3D) HT-29 cells cultured under control, moderate low Mg^2+^ (0.4 mM; low-Mg), and moderate high Mg^2+^ (2.5 mM; high-Mg) conditions underwent TRPM6 immunoprecipitation. (**a**) Representative blots and quantification of immunoprecipitated TRPM6, confirming uniform loading. (**b**) Representative blots and quantification of TRPM7 co-immunoprecipitated with TRPM6, reflecting TRPM6/7 heterodimer abundance. (**c**) 3D immunofluorescence images of SPs stained for TRPM6 (green), TRPM7 (red), and nuclei (Hoechst, blue), with a yellow signal indicating TRPM6–TRPM7 co-localization. Scale bar: 100 µm. (**d**) Representative blots and quantification of Mrs2 protein expression. Data are mean ± SD (n = 5). Statistical analysis was performed using one-way ANOVA with Dunnett’s post hoc test for multiple comparisons versus control groups and unpaired Student’s *t*-test for comparisons between parental and SP groups under the same Mg^2+^ condition. The significance levels are as follows: * *p* < 0.05, ** *p* < 0.01, *** *p* < 0.001 vs. control parental group. † *p* < 0.05, †† *p* < 0.01 vs. parental cells under matched Mg^2+^ conditions. ### *p* < 0.001 vs. control SPs.

The channel permeability of TRPM6 and TRPM7 is suppressed by intracellular Mg^2+^ and Mg·ATP [23,24]. Critically, heterodimeric TRPM6/7 complexes are comparatively resistant to this inhibition [23,24], enabling sustained directional Mg^2+^ absorption. To determine whether impaired Mg^2+^ influx and reduced viability under Mg^2+^ stress were linked to reduced channel dimerization at the plasma membrane, we immunoprecipitated TRPM6 (IP-TRPM6) from membrane fractions and analyzed associated proteins by Western blot. Comparable TRPM6 levels in IP fractions confirmed uniform loading (Figure 7a). Subsequent reprobing for TRPM7 revealed a markedly higher TRPM6/7 association in control SPs compared to parental cells, indicating elevated basal heterodimerization (Figure 7b). Both low and high Mg^2+^ conditions significantly decreased co-precipitated TRPM7 in SPs, demonstrating loss of membrane-bound heterodimers. Under identical Mg^2+^ conditions, SPs consistently exhibited weaker TRPM7 signals than parental cells, confirming their greater susceptibility to Mg^2+^-dependent destabilization of TRPM6/7 complexes. We further illustrated this disruption using high-resolution imaging with the ZEISS LSM910 Lightfield 4D microscope (Figure 7c): control SPs displayed strong co-localization of TRPM6 (green) and TRPM7 (red). This peri-spheroidal expression of TRPM6, TRPM7, and their heterodimeric complexes was markedly reduced in both low and high Mg^2+^ conditions, visually corroborating the biochemical findings.

### 2.6. Mg^2+^ Dysregulation Preferentially Suppresses the Mitochondrial Mg^2+^ Importer Mrs2 in SPs

The mitochondrial Mg^2+^ channel Mrs2 is a central determinant of mitochondrial Mg^2+^ homeostasis and thereby of cellular bioenergetics and survival [25], with its expression inversely associated with stress-induced cell death [26]. We therefore examined Mrs2 regulation under Mg^2+^ stress in our CRC models. SPs, which display elevated metabolic activity and greater viability, exhibited significantly higher basal Mrs2 levels than parental cells (Figure 7d). Both low and high Mg^2+^ conditions significantly suppressed Mrs2 expression in each model. This suppression was consistently stronger in SPs compared to the same Mg^2+^ conditions, paralleling their enhanced susceptibility to cell death. These findings implicate impaired mitochondrial Mg^2+^ import via Mrs2 as a key driver of metabolic failure in Mg^2+^-stressed SPs. The amplified loss of Mrs2 provides a direct mitochondrial mechanism underlying the heightened vulnerability and extensive cell death induced by Mg^2+^ dysregulation in the SP phenotype.

### 2.7. Nano-LC-MS/MS Analysis of Membranous TRPM6 and TRPM7

To characterize the composition of Mg^2+^ transport complexes, we conducted nano-liquid chromatography tandem mass spectrometry (nano-LC-MS/MS) on membrane proteins isolated through TRPM6 immunoprecipitation [13,27]. Sequence analysis verified TRPM6 (UniProtKB: Q9BX84) and its partner TRPM7 (UniProtKB: Q96QT4) in all groups, and comparable exponentially modified protein abundance index (emPAI) values (TRPM6: parental 0.10, SP 0.12, SP + low-Mg 0.11, SP + high-Mg 0.10; TRPM7: parental 0.11, SP 0.11, SP + low-Mg 0.12, SP + high-Mg 0.10) confirmed equivalent loading.

We then examined functionally significant phosphorylation sites relevant to channel function. Particular attention was given to TRPM6 Ser141 (S141), a key determinant of heterodimer membrane localization [28]. S141 phosphorylation was detected in parental cells and control SPs but was lost entirely under both low and high Mg^2+^ conditions (Table 1), providing a mechanistic explanation for reduced TRPM6/7 heterodimer. Additionally, regulatory sites governing channel gating were also identified. Phosphorylation of S1252, a modification that increases permeability [29], occurred exclusively in control SPs, consistent with their elevated Mg^2+^ influx, and was absent in parental cells and Mg^2+^-stressed SPs. In contrast, phosphorylation of T1851, a known inhibitory site [30], was detected only in low and high Mg^2+^ SPs, confirming active suppression of Mg^2+^ influx under stress.

**Figure 8:**
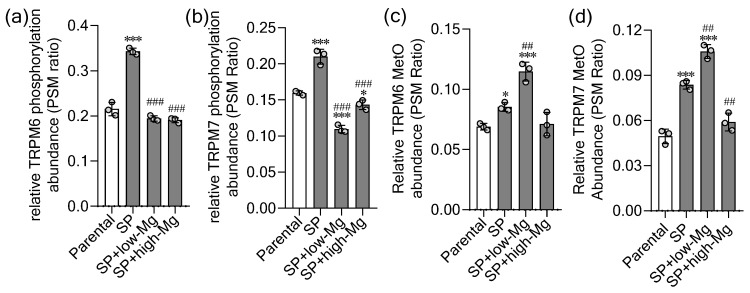
Proteomic analysis of post-translational modifications in TRPM6 and TRPM7 under Mg^2+^ dysregulation. The relative abundance of (**a**) TRPM6 phosphorylation, (**b**) TRPM7 phosphorylation, (**c**) TRPM6 methionine oxidation (MetO), and (**d**) TRPM7 MetO was determined by peptide spectrum match (PSM) ratios in parental (2D) and spheroid (SP)-derived (3D) HT-29 cells. Cells were cultured under control, moderate low Mg^2+^ (0.4 mM; low-Mg), and moderate high Mg^2+^ (2.5 mM; high-Mg) conditions. Data are presented as mean ± standard deviation (SD) (n = 3). Statistical significance was assessed by one-way ANOVA followed by Dunnett’s post hoc test for multiple comparisons versus the respective control group (2D or 3D). * *p* < 0.05, *** *p* < 0.001 versus the control parental group; ## *p* < 0.01, ### *p* < 0.001 versus the control SP group.

Globally, the total number of phosphorylated TRPM6 residues paralleled Mg^2+^ influx capacity [13]: 200 in control SPs (high influx) versus 171, 172, and 164 in parental, low Mg^2+^, and high Mg^2+^ groups (reduced influx), respectively. The relative abundance of these phosphorylated residues further confirmed distinct phosphoproteomic patterns across all experimental conditions (Figure 8a). These phosphoproteomic patterns reveal how non-physiological Mg^2+^ levels impair channel function by reshaping TRPM6’s phosphorylation landscape, altering its membrane localization and permeability.

We extended our phosphoproteomic analysis to TRPM7 and identified regulatory sites that closely mirrored the TRPM6 profile. Phosphorylation of S138, which governs TRPM7 membrane localization [28], and S1360, which stabilizes its plasma membrane presence [31], was detected in parental cells and control SPs but disappeared in Mg^2+^-stressed SPs (Table 2), consistent with reduced membranous TRPM7.

A global survey further showed that TRPM7 carried the highest number of phosphorylated residues in control SPs (153), followed by parental cells (138) and high Mg^2+^ SPs (136), with low Mg^2+^ SPs exhibiting the fewest (107). The relative abundance of these phosphorylated sites confirmed distinct condition-specific phosphoproteomic signatures (Figure 8b). This phosphorylation burden on TRPM7 correlated positively with Mg^2+^ influx across all groups [13].

Together, these phosphoproteomic findings define a unified mechanism in which non-physiological Mg^2+^ environments disrupt TRPM6/7 heterodimers by reconfiguring the phosphorylation states of both subunits, thereby modulating their membrane localization, stability, and channel permeability.

Our MS/MS analysis also revealed extensive methionine oxidation (MetO) across TRPM6 and TRPM7 (Table 3). We concentrated on the M1755 residue of TRPM6, as its oxidation is known to impede channel function [32]. Consistent with this mechanism, M1755 oxidation was strongly enriched in SPs exposed to low and high Mg^2+^, providing a direct post-translational basis for their reduced Mg^2+^ influx.

Global MetO was likewise elevated, with the low Mg^2+^ group exhibiting the highest number of oxidized residues (87) relative to control SPs (70), parental cells (57), and high Mg^2+^ SPs (64), indicating substantial Mg^2+^-deprivation-induced oxidative stress on channel proteins. This pronounced elevation under low Mg^2+^ condition indicates substantial oxidative stress on channel proteins induced by Mg^2+^ deprivation. The relative abundance of these oxidized residues confirmed distinct, condition-specific oxidation signatures for both TRPM6 and TRPM7 (Figure 8c,d).

Collectively, these phospho- and oxidoproteomic findings delineate a dual mechanism by which non-physiological Mg^2+^ conditions destabilize the TRPM6/7 heterodimer: perturbed phosphorylation reshapes membrane localization and permeability, and at the same time, oxidative modifications further diminish channel activity.

## 3. Discussion

Dysregulated cellular Mg^2+^ homeostasis in CRC is well-established [6,7,8,9,10,11,12,13]. Our study advances this understanding by clarifying the mechanisms involved using a three-dimensional SP model. Consistent with CRC tissue observations [6], SPs displayed markedly elevated intracellular Mg^2+^ relative to parental cells. This increase stemmed from enhanced Mg^2+^ influx driven by upregulated total and membrane-bound TRPM6, TRPM7, and TRPM6/7, together with reduced expression of the efflux transporter CNNM4. The increased Mg^2+^ influx in SPs seems to be related to the higher number of membrane-localized TRPM6/7 heterodimers. These complexes exhibit diminished sensitivity to inhibition by intracellular Mg^2+^ and Mg·ATP, allowing for persistent Mg^2+^ uptake [23,24]. Our proteomic data offer direct molecular support for this activated state. In control SP, we identified TRPM6 phosphorylation at Ser141 (promoting heterodimerization [28]) and Ser1252 (increasing channel permeability [29]), along with TRPM7 phosphorylation at Ser138 (regulating membrane localization [28]) and Ser1360 (stabilizing plasma membrane residency [31]). This distinct phospho-pattern likely defines the constitutive Mg^2+^-acquisitive phenotype of CRC SPs.

The heightened viability of the SP phenotype reflects a multifaceted pro-survival program defined by increased Mg^2+^ content, elevated expression of the mitochondrial Mg^2+^ channel Mrs2, and high levels of the anti-apoptotic protein Bcl-2. Mrs2-dependent mitochondrial Mg^2+^ uptake is crucial for efficient ATP synthesis and serves as a physiological inhibitor of the mitochondrial calcium uniporter (MCU) [33]. By restricting MCU activity, elevated intramitochondrial Mg^2+^ prevents Ca^2+^ overload, thereby blocking activation of the mitochondrial permeability transition pore (MPTP) [34], a process that induces ΔΨm collapse, halts ATP synthesis, and triggers apoptosis [34]. Consequently, the high basal Mg^2+^ and Mrs2 levels in SPs establish a protective mitochondrial environment. This is supported by their markedly higher ΔΨm, a direct measure of the proton gradient magnitude and coupled ATP-generating capacity [26]. This enhanced ΔΨm, together with the reported ability of increased Mg^2+^/Mrs2 to suppress caspase activity [35], renders the SP phenotype intrinsically apoptosis-resistant and confers protection against inducers such as doxorubicin (DXR) and staurosporine (STS) [12,26]. Complementing this mitochondrial defense, SPs displayed elevated basal Bcl-2 expression. In cancer cells, Bcl-2 overexpression blocks mitochondrial translocation and activation of pro-apoptotic Bax, thereby directly inhibiting the intrinsic apoptotic cascade and promoting resistance to agents like STS [36]. Thus, the integrated profile of elevated Mg^2+^ influx, increased Mrs2, enhanced ΔΨm, and upregulated Bcl-2 generates a potent state of augmented energy metabolism and apoptotic resistance under physiological conditions. This strong basal framework not only reinforces the validity of our model but also highlights the profound cytotoxic consequences that arise when Mg^2+^ dysregulation perturbs this finely balanced system.

Our investigation indicates that the SP phenotype, despite its strong baseline, displays heightened sensitivity to extracellular Mg^2+^ dysregulation compared with parental cells. This vulnerability was reflected in the marked loss of viability and the amplified activation of apoptotic and autophagic markers under low and high Mg^2+^ conditions. We propose that this hypersensitivity arises from the convergence of metabolic and oxidative stresses. First, an Mg^2+^ imbalance is known to trigger cellular and mitochondrial oxidative damage capable of directly initiating cell-death pathways [37]. Although we did not directly assess reactive oxygen species, our oxidoproteomic data provide persuasive indirect evidence: SPs exposed to Mg^2+^ stress showed a substantially higher number of oxidatively modified methionine residues (Table 3), indicating increased protein oxidation. Second, the pronounced loss of ΔΨm in stressed SPs indicates a profound failure of energy metabolism, likely impairing ATP production. Thus, the SP’s sensitivity appears rooted in an inability to withstand the dual pressures of Mg^2+^ imbalance: (1) an elevated oxidative load demonstrated by protein damage and (2) a collapse of mitochondrial bioenergetics. This combination likely produces an irreversible metabolic breakdown, ultimately driving the extensive cell death observed.

Our previous work showed that pharmacological inhibition of TRPM6 and TRPM6/7 suppresses Mg^2+^ content, thereby impairing the stemness, progression, and metastatic capacity of HT-29 SPs [13]. The present study expands on these findings by evaluating the impact of both physiological extremes—Mg^2+^ deprivation (low Mg^2+^) and Mg^2+^ excess (high Mg^2+^). This represents a novel contribution, as previous studies focused on pharmacological inhibition rather than the physiological consequences of Mg^2+^ imbalance. Our results reveal a nuanced and context-dependent toxicity profile. Although low Mg^2+^ condition significantly impaired SP progression, high Mg^2+^ exerted an even more disruptive influence across several parameters, including a sharper reduction in SP area and density, lower viability, and greater induction of p53, cleaved caspase-3, pS556 ULK1, and p62/SQSTM1. These findings indicate that supra-physiological Mg^2+^ levels are not merely inhibitory but can provoke a more severe stress response, leading to accelerated metabolic and structural collapse. Thus, both removal of the essential ion and its pathological excess effectively compromise CRC SP integrity, with high Mg^2+^ condition demonstrating superior potency in eliciting specific hallmarks of programmed cell death.

**Figure 9:**
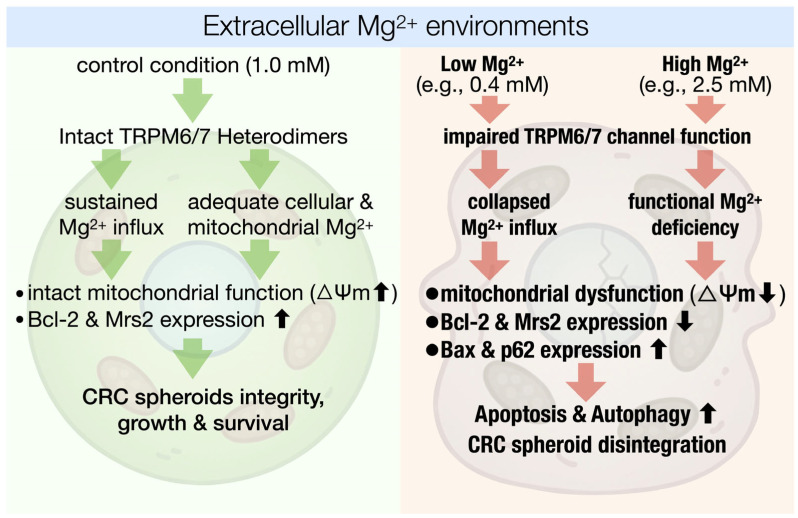
A unified model of Mg^2+^-dependent fate determination in colorectal cancer spheroids. This schematic summarizes the divergent cellular and metabolic outcomes in HT-29 spheroids cultured under varying extracellular Mg^2+^ concentrations. Under physiological control conditions (1.0 mM Mg^2+^), intact TRPM6/7 heterodimers sustain Mg^2+^ influx, ensuring adequate cellular and mitochondrial Mg^2+^ content, maintained ΔΨm, and enhanced expression of anti-apoptotic (Bcl-2) and mitochondrial Mg^2+^-import (Mrs2) proteins. Together, these processes support spheroid integrity, growth, and survival. Both low (0.4 mM) and high (2.5 mM) extracellular Mg^2+^ environments disrupt Mg^2+^ homeostasis through distinct initial mechanisms that converge on a common pathogenic endpoint: Mg^2+^ influx collapse, mitochondrial Mg^2+^ deficiency, loss of ΔΨm, and downregulation of Bcl-2 and Mrs2. These changes trigger upregulation of pro-apoptotic (Bax) and autophagic (p62) markers, leading to increased apoptosis and autophagy, ultimately resulting in spheroid disintegration. The model underscores the dual vulnerability of colorectal cancer spheroids to Mg^2+^ dysregulation and highlights the TRPM6/7–Mrs2 axis as a central node for therapeutic intervention.

Low Mg^2+^ conditions, paralleling the effects of TRPM6/7 inhibition [13], promoted CRC SP degradation and death through intracellular Mg^2+^ depletion. Reduced extracellular Mg^2+^ lowered the driving force for Mg^2+^ influx through TRPM6, TRPM7, and their heterodimers, as directly evidenced by our Mg^2+^ influx assays. This process led to a loss of both free and total cellular Mg^2+^. Coupled with decreased Mrs2 expression, this deficit resulted in mitochondrial Mg^2+^ depletion [37]. The ensuing loss of Mg^2+^-mediated the MCU inhibition, which activated the MCU [33,37], triggering Bcl-2 downregulation, ΔΨm collapse, and the induction of apoptotic and autophagic pathways [34,37], ultimately leading to the extensive cell death observed (Figure 9). Translating this mechanism into therapy remains challenging; inducing systemic hypomagnesemia is clinically unfeasible due to severe neuromuscular and cardiac risks. A more practical approach would involve localized Mg^2+^ channel blockade within the TME. This could be achieved by delivering TRPM6/7 inhibitors with complementary pro-apoptotic agents through controlled-release systems, such as microsphere-encapsulated formulations targeted to the colon or rectum. Such an approach would exploit the Mg^2+^-dependence of CRC SPs while avoiding the systemic liabilities associated with whole-body Mg^2+^ depletion.

High Mg^2+^ condition produces a more potent toxic effect on CRC SPs than low Mg^2+^, as shown by sharper reductions in SP area, structural integrity, and viability, along with stronger induction of p53, cleaved caspase-3, p62, and ULK1, and a more pronounced suppression of Mrs2 expression and ΔΨm (Figure 8). We propose a distinct mechanism underlying high Mg^2+^ toxicity. Although total cellular Mg^2+^ increases, the free intracellular Mg^2+^ pool in SPs is paradoxically reduced (Figure 5c), likely reflecting Mg^2+^ buffering and sequestration. The permeability of membrane TRPM6/7 channels is significantly inhibited when intracellular Mg^2+^ and Mg·ATP levels increase [24]. The elevated total Mg^2+^ under high Mg^2^ may therefore directly suppress TRPM6/7 activity, accounting for the markedly impaired Mg^2+^ influx (Figure 5b). This process initiates a detrimental cycle: (1) inhibited influx prevents restoration of the free cytosolic Mg^2+^ pool; and (2) reduced Mrs2 expression (Figure 8) restricts mitochondrial Mg^2+^ import. As a result, despite elevated total Mg^2+^, the SP experiences a functional deficit in the bioavailable Mg^2+^ needed for metabolic support and anti-apoptotic signaling. This “functional Mg^2+^ depletion” at key regulatory sites (cytosol, mitochondria) disturbs bioenergetics (low ΔΨm) and activates cell-death pathways, driving the extensive SP disintegration observed.

### 3.1. Interacting Pathways and Broader Biological Context

While our study focused on the TRPM6/7-Mg^2+^ axis, it is important to acknowledge that Mg^2+^ homeostasis interacts with multiple signaling pathways that could contribute to the observed effects. Reactive oxygen species (ROS) generation is known to be modulated by Mg^2+^ status [37], and oxidative stress could exacerbate channel dysfunction and mitochondrial damage. Additionally, calcium signaling through the mitochondrial calcium uniporter (MCU) is directly inhibited by Mg^2+^ [33], creating cross-talk between these two essential divalent cations. The mTOR pathway, which regulates autophagy and cell growth, is also sensitive to Mg^2+^ availability [38]. Future studies should explore these interactions to provide a more comprehensive understanding of Mg^2+^’s role in CRC progression.

Our findings in CRC SPs contrast with some reports in other cell types, highlighting the context-dependent nature of Mg^2+^ biology. The intestinal-specific expression of TRPM6 and functional TRPM6/7 heterodimers [15] provides a unique molecular basis for the distinct Mg^2+^ handling we observed in HT-29 SPs. For instance, in bone marrow mesenchymal stem cell (BM-MSC) SPs, which likely rely on a different repertoire of Mg^2+^ channels, elevated Mg^2+^ was shown to promote proliferation and upregulate MagT1 expression [39]. Conversely, in our HT-29 SPs, elevated Mg^2+^ downregulated MagT1 and inhibited growth. This fundamental discrepancy may stem not only from differences between normal stem cell and transformed cancer cell metabolism but also from the cell-type-specific Mg^2+^ influx machinery, where the intestinal TRPM6/7 system in CRC is co-opted to support distinct, potentially more rigid oncogenic programs that are uniquely vulnerable to dysregulation.

Similarly, the relationship between Mg^2+^ and oxidative stress appears to be finely tuned by cellular context. While Mg^2+^ deficiency consistently increases ROS production across models, as demonstrated in chick embryo hepatocytes [40], the effect of high Mg^2+^ is variable. In human keratinocytes, Mg^2+^ supplementation up to 5 mM was shown to attenuate H_2_O_2_-induced mitochondrial damage and cell death, likely through a protective release of Mg^2+^ from ATP [41]. In stark contrast, our model suggests that similarly elevated Mg^2+^ induces a state of functional Mg^2+^ deficiency and metabolic collapse in CRC SPs, associated with increased protein oxidation. This opposing outcome underscores that the cellular response to Mg^2+^ is not linear and is critically shaped by the underlying metabolic landscape and, importantly, the specific molecular pathways (such as TRPM6/7) that govern cellular Mg^2+^ homeostasis.

Unlike systemic hypomagnesemia, inducing a localized high Mg^2+^ TME through controlled-release systems is a feasible and promising therapeutic strategy. Mg-based biomaterials or microsphere-encapsulated hydrogels can deliver sustained, spatially confined Mg^2+^ release [17,18]. Such an approach could exploit the SP’s susceptibility to Mg^2+^ overload, triggering the toxic cycle described above, while leveraging the immunomodulatory properties of Mg^2+^ to enhance treatments such as immune checkpoint blockade [17]. The biodegradability and biocompatibility of Mg-based implants make them especially suitable for this purpose [18], highlighting the therapeutic potential of precisely modulating tumoral Mg^2+^ fluxes.

### 3.2. Limitations

This study has several limitations that should be acknowledged. First, we used a single colorectal cancer cell line (HT-29), which may not fully represent the heterogeneity of CRC. Future studies should include additional CRC cell lines and patient-derived organoids. Second, our findings are based entirely on in vitro 3D SP models, which, while more physiologically relevant than 2D cultures, still lack the complexity of in vivo TME. The absence of immune cells, stromal components, and vascularization limits direct translation to clinical settings. Third, we did not measure ROS directly, although our oxidoproteomic data suggest increased oxidative stress. Fourth, the study design does not establish causality but rather demonstrates associations between Mg^2+^ dysregulation and cellular outcomes. Finally, we focused primarily on the TRPM6/7-Mrs2-Bcl-2 axis without fully exploring other potential mediators of Mg^2+^-induced cell death. These limitations highlight the need for future in vivo validation and more comprehensive mechanistic studies.

## 4. Materials and Methods

### 4.1. Cell Culture

HT-29 cells (ATCC HTB-38) were cultured in Dulbecco’s modified Eagle medium (DMEM; Gibco, Waltham, MA, USA) supplemented with 15% fetal bovine serum, 1% L-glutamine, 1% nonessential amino acids, and 1% penicillin-streptomycin (all from Gibco). All experiments used early-passage cells (passages 5–15) that were not exposed to prior treatments, ensuring consistent metabolic status. Cells were maintained under standard conditions at 37 °C in a humidified atmosphere containing 5% CO_2_. Subculturing was performed in 75 cm^2^ tissue culture flasks (Corning Inc., Corning, NY, USA) following the ATCC guidelines.

### 4.2. SP Formation and Magnesium Modulation

SP formation was triggered by culturing HT-29 cells under nonadherent, serum-free conditions in a 96-well hanging-drop plate (Perfecta3D^®^, Sigma-Aldrich, Saint Louis, MO, USA). A total of 5 × 10^4^ cells/well were seeded in DMEM/F-12 medium containing 1 × B27 supplement, 20 ng/mL EGF, and 20 ng/mL bFGF (all from PeproTech, Cranbury, NJ, USA). The reservoir was filled with 5 mL PBS to prevent evaporation. Cultures were maintained for up to 14 days at 37 °C and 5% CO_2_, during which cells aggregated into compact, uniform SPs. Morphological analysis was performed using AnaSP version 3.0 as previously described [13].

To generate controlled Mg^2+^ environments, SPs were washed with Ca^2+^/Mg^2+^-free Dulbecco’s Phosphate-Buffered Saline (DPBS; HiMedia Laboratories, Mumbai, India) and subsequently transferred to a custom HyClone™ DMEM base lacking Ca^2+^, Mg^2+^, L-glutamine, and sodium pyruvate (Cytiva, Marlborough, MA, USA). This base was supplemented with 15% FBS, 1% L-glutamine, 1% non-essential amino acids, and 1.25 mM CaCl_2_. Sterile MgCl_2_ was added to achieve the following final concentrations:

- Control (physiological): 1.0 mM Mg^2+^ (based on normal serum Mg^2+^ levels [15])
- Low Mg^2+^ conditions: 0.2 mM (severe), 0.4 mM (moderate), 0.6 mM (mild) Mg^2+^ (representing clinically relevant deficiency levels [15])
- High Mg^2+^ conditions: 1.5 mM (mild, mimicking moderate clinical hypermagnesemia), 2.5 mM (moderate, mimicking symptomatic clinical hypermagnesemia), 5.0 mM (severe, mimicking severe clinical hypermagnesemia) Mg^2+^ (supraphysiological levels used in experimental models, based on clinical thresholds [15])

All media were verified to fall within physiological pH and osmolarity ranges.

### 4.3. Cell Viability Assay (WST-1)

SP viability was quantified using the WST-1 cell proliferation reagent (Sigma-Aldrich, Cat. No. 05015944001), which reports mitochondrial dehydrogenase activity. After treatment, SPs were exposed to WST-1 for 4 h at 37 °C to allow enzymatic conversion of the tetrazolium substrate to formazan. Absorbance was measured using an EnSight™ multimode plate reader (PerkinElmer, Waltham, MA, USA) at 450 nm with 620 nm as reference. Each experiment included blank controls (medium without cells) and negative controls (untreated cells). Data were normalized to control conditions (set as 100% viability).

### 4.4. Mg^2+^ Influx Measurements

For Mg^2+^ influx analysis, subconfluent cells grown in μ-Dishes (Ibidi, Gräfelfing, Germany) were preincubated in DMEM for 3 days to equilibrate baseline transport activity. On the assay day, cells were rinsed with Ca^2+^/Mg^2+^-free DPBS (HiMedia Laboratories, Thane, India) and incubated for 1 h in a custom HyClone™ DMEM base devoid of Ca^2+^, Mg^2+^, L-glutamine, and sodium pyruvate (Cytiva, Marlborough, MA, USA), supplemented with 15% FBS, 1% L-glutamine, and 1% non-essential amino acids. Cells were then loaded with 5 μmol/L Mag-Fura-2-AM (Invitrogen, Waltham, MA, USA) in N-methyl-D-glucamine buffer for 1 h, followed by a 30 min de-esterification in dye-free buffer. Real-time fluorescence imaging was performed on an Olympus FV3000 confocal microscope (Olympus Corporation, Tokyo, Japan). Mg^2+^ influx was calculated as ΔF/F = [F(t) − F(0)]/[F(0) − F(b)], where F(t) is the mean fluorescence intensity at a given time, F(0) is the basal fluorescence intensity, and F(b) is the background fluorescence. Image analysis was conducted using ImageJ (https://imagej.net/ij/download.html accessed on 11 December 2025) [13].

### 4.5. Analysis of Mitochondrial Membrane Potential (ΔΨm)

ΔΨm was evaluated in parental and SP-derived cells using the JC-1 Mitochondrial Membrane Potential Assay Kit (Abcam, Cambridge, UK, cat. no. ab113850). The assay was conducted according to the manufacturer’s instructions, with modifications based on the work of Anaya-Eugenio et al. [42]. Briefly, cells were seeded in 96-well plates and allowed to adhere before incubation with JC-1 working solution for 20 min at 37 °C in the dark. After staining, cells were gently rinsed with warm PBS to remove excess dye. Fluorescence was recorded using an EnSight™ multimode plate reader (PerkinElmer). JC-1 exhibits potential-dependent mitochondrial accumulation, as reflected by an emission shift from green (~530 nm; monomers, low ΔΨm) to red (~590 nm; aggregates, high ΔΨm). The red-to-green fluorescence ratio was calculated to quantify mitochondrial polarization. Control wells with FCCP (carbonyl cyanide-p-trifluoromethoxyphenylhydrazone) were included to confirm depolarization.

### 4.6. Measurement of Intracellular Mg^2+^ Levels

Free Mg^2+^: Subconfluent cells in 6-well plates were preincubated for 3 days, then loaded with 5 μmol/L Mag-Fura-2-AM in standard bathing solution [13] for 30 min at 37 °C, followed by a 30 min de-esterification period. Fluorescence was acquired using an Olympus FV3000 confocal microscope to quantify cytosolic free Mg^2+^.

Total Mg^2+^: Total cellular Mg^2+^ content was measured by atomic absorption spectrophotometry (Shimadzu, Kyoto, Japan). Cells were lysed by sonication, and Mg^2+^ concentrations were determined relative to a standard curve, as per the standard protocols [13].

For both measurements, blank samples (no cells) and standard solutions were included for calibration.

### 4.7. Immunoprecipitation

TRPM6 immunoprecipitation (IP) was carried out according to established procedures [13,27]. Cells were lysed in cold RIPA buffer containing protease and phosphatase inhibitors, and membrane fractions were isolated with the Mem-PER™ Plus kit (Thermo Fisher Scientific, Waltham, MA, USA). Lysates were incubated overnight at 4 °C with an anti-TRPM6 polyclonal antibody (Thermo Fisher Scientific). Afterward, immune complexes were captured using Protein A/G Sepharose^®^ beads (Abcam). Following rigorous washing, bound proteins were eluted with glycine–Tris buffer (Sigma-Aldrich) and concentrated using Vivaspin^®^ 20 centrifugal filters (Sartorius AG, Göttingen, Germany) for subsequent analysis. Control IPs with normal rabbit IgG were performed to assess non-specific binding.

### 4.8. Western Blot Analysis

Western blotting was performed according to standard protocols [13,27]. Protein samples were resolved by SDS–PAGE and electrotransferred onto nitrocellulose membranes. Precision Plus Protein™ Dual Xtra Prestained Standards (Cat. No. 1610377; Bio-Rad Laboratories, Hercules, CA, USA) served as molecular weight markers. Membranes were probed with specific primary antibodies against p53 (PB9008; Bosterbio, Pleasanton, CA, USA), BCL-2 (A00040; Bosterbio), Bax (A00183; Bosterbio), caspase-3 (PB9188; Bosterbio), cleaved caspase-3 (ab32042; Abcam), pS556 ULK1 (ab203207; Abcam), p62/SQSTM1 (ab109012; Abcam), TRPM6 (0ST00108W; Thermo Fisher Scientific Inc.), TRPM7 (ab729; Abcam), MagT1 (ab244490; Abcam), CNNM4 (ab191207; Abcam), Mrs2 (ab246915; Abcam), β-actin (ab6276; Abcam), and Na^+^/K^+^-ATpase (ab76020; Abcam). After incubation with HRP-conjugated secondary antibodies, protein bands were detected using SuperSignal^®^ West Pico chemiluminescent substrate (Thermo Fisher Scientific) and imaged with a ChemiDoc™ Touch system (Bio-Rad Laboratories). Densitometric quantification was performed with ImageJ software. Protein expression levels were normalized to the parental control group. β-actin served as the loading control for total cell lysates and cytosolic fractions, while Na^+^/K^+^-ATPase was used for membrane fractions.

### 4.9. Immunofluorescence

For TRPM6 and TRPM7 localization, SPs were fixed in 4% paraformaldehyde for 10 min at room temperature and then permeabilized in pre-chilled methanol (−20 °C) for 10 min. Blocking was performed sequentially with 0.1% glycine for 10 min, followed by a 30 min incubation in a protein-free blocking buffer (Visual Protein, Neihu Dist., Taipei, Taiwan). Primary antibodies were diluted 1:100 in blocking buffer and applied overnight at 4 °C. After washing, SPs were treated with fluorophore-conjugated secondary antibodies (1:500; FITC, ab6717; or Alexa Fluor^®^ 568, ab175474; Abcam) for 1 h at room temperature. Nuclei were counterstained with Hoechst (Molecular Probes, Eugene, OR, USA) according to the manufacturer’s guidelines. Fluorescent images were acquired using the ZEISS LSM910 Lightfield 4D microscope (Carl Zeiss Microscopy GmbH, Jena, Germany). Negative controls (no primary antibody) were included to assess non-specific staining.

### 4.10. Nano-Liquid Chromatography Tandem Mass Spectrometry Analysis (nanoLC-MS/MS)

Proteomic profiling of membranous TRPM6 and TRPM7 was performed by nanoLC-MS/MS as previously described [27]. To minimize artifactual methionine oxidation (MetO), all steps were conducted rapidly using fresh reagents. Proteins were purified (Clean-up kit, GE Healthcare, Chicago, IL, USA), solubilized in 8 M urea, and quantified via Bradford assay (Bio-Rad Laboratories, Hercules, CA, USA). For in-solution digestion, 20 µg of protein was reduced (100 mM DTT in 100 mM TEAB, 30 min, RT), alkylated (100 mM iodoacetamide, 30 min, dark), and digested with trypsin (Trypsin Gold, Promega, Madison, WI, USA; 1:50 *w*/*w*, 16 h, 37 °C). Peptides were desalted (C18 Zip-tips, MilliporeSigma), dried, and stored at −80 °C.

Prior to LC-MS/MS, peptides were reconstituted in 0.1% formic acid. Separations were performed on a Dionex Ultimate 3000 RSLCnano system coupled to a Q-ToF Compact II mass spectrometer (Bruker Daltonics, Billerica, MA, USA). Peptides (1 µg) were loaded onto a PepMap100 C18 column (75 µm × 500 mm, 3 µm) and eluted over a 90 min linear gradient (2–95% solvent B: 80% acetonitrile, 0.08% FA) at 300 nL/min. Data were acquired in positive-ion, data-dependent acquisition mode (m/z 150–2200) with collision-induced dissociation (CID).

Raw files were processed using DataAnalysis software (Bruker Daltonics, version 5.3) and searched against the UniProtKB human reference proteome database (release 2023_01) using the MASCOT search engine (v2.3; Matrix Science, London, UK). Search parameters specified: trypsin/P digestion with up to two missed cleavages; precursor and fragment mass tolerances of 20 ppm and 0.05 Da, respectively; fixed modification of carbamidomethylation (C); variable modifications of MetO, N-terminal acetylation, and phosphorylation (Ser, Thr, Tyr). Protein and peptide identifications were validated at a false discovery rate (FDR) of <1%. The exponentially modified protein abundance index (emPAI) was used for semi-quantitative protein abundance comparison.

To specifically address changes in post-translational modifications (PTMs), the relative abundance of MetO for key residues and phosphorylation for key regulatory sites was determined as previously described [43]. The extracted ion chromatogram (XIC) peak area for the modified peptide form was compared to the peak area of its non-modified counterpart identified in the same LC-MS/MS run. The peptide spectrum match (PSM) ratios were calculated for each sample, providing a relative measure of phosphorylation and oxidation status across experimental groups. All samples were analyzed in triplicate (technical replicates, n = 3).

### 4.11. Limitations

Our mass spectrometry analysis, while providing novel insights into TRPM6/7 modifications, has inherent constraints. Phosphopeptide enrichment was not employed; therefore, our identification of phosphorylation sites, though validated, may not be comprehensive for low-abundance phosphopeptides. The quantification of post-translational modifications was focused on MetO via XIC-based ratios; phosphorylation sites were identified but not similarly quantified due to the lack of enrichment and lower spectral counts. Although stringent precautions were taken, the reported MetO levels represent relative changes between groups and could include minor artifactual oxidation.

### 4.12. Statistical Analysis

Data are expressed as mean ± standard deviation (SD). Data normality was confirmed using the Shapiro–Wilk test. Differences between two groups were analyzed using an unpaired Student’s *t*-test (used for comparisons between parental and SP groups under the same Mg^2+^ condition), whereas comparisons across multiple groups employed one-way analysis of variance with Dunnett’s post hoc test for multiple comparisons versus control groups. A *p*-value < 0.05 was considered statistically significant. All statistical analyses were performed using GraphPad Prism 8.0.1 (GraphPad Software Inc., San Diego, CA, USA).

## 5. Conclusions

Our in vitro study demonstrates that CRC spheroids, while displaying a Mg^2+^-acquisitive phenotype that supports growth under physiological conditions, harbor a critical vulnerability: their dependence on tightly regulated Mg^2+^ homeostasis makes them acutely sensitive to both depletion and overload of this essential ion. We further clarify the mechanistic basis of this sensitivity, showing that departures from physiological Mg^2+^ disrupt the core regulatory apparatus—chiefly the TRPM6/7 heterodimer—through post-translational modifications that diminish membrane localization and channel conductance. This results in a progressive collapse of Mg^2+^ handling, mitochondrial impairment, and the activation of coordinated apoptotic and autophagic cell-death programs. Notably, high Mg^2+^ stress proved especially potent, inducing a form of “functional Mg^2+^ depletion” that drives profound metabolic failure. These findings shift the paradigm from viewing Mg^2+^ merely as a pro-tumorigenic factor to recognizing its dysregulation as a compelling therapeutic target. However, the in vitro nature of this study and use of a single cell line necessitate cautious interpretation and future in vivo validation. They offer strong justification for developing localized strategies, such as controlled-release Mg^2+^ formulations or selective channel inhibitors, that intentionally disrupt intratumoral Mg^2+^ homeostasis to hinder CRC progression while limiting systemic toxicity. Our results suggest that both deficiency and excess of Mg^2+^ can be exploited therapeutically, with high Mg^2+^ showing particular promise for localized intervention. The “dividing line” between beneficial and harmful Mg^2+^ levels appears to depend on cellular adaptive capacity rather than a fixed concentration, highlighting the importance of context-specific modulation.

## Figures and Tables

**Table 1:** Phosphorylated residues of TRPM6 protein.

	Parental	SP	SP + Low-Mg	SP + High-Mg
N-terminus	S12 S67 S78 Y80 T87 T88 S90 T92 T94 **S141** T169 T170 T176 T181 Y228 S243 S246 S274 S282 S302 T380 S386 T398 S407 Y431 S445 Y462 Y479 T481 T509 T530 S552 S558 Y574 S586 S596 T602 Y608 T635 S662 Y697 S705 T706 S743 T756 S820 Y835 Y845 T859 S869	S12 S32 S78 Y80 T87 T88 T94 Y111 Y116 **S141** S153 T169 T170 T176 T181 S184 T204 Y228 T230 S236 T239 Y259 S274 S282 T354 S362 T380 S407 S409 Y431 S445 T471 Y506 T509 Y519 Y525 Y529 T530 Y545 S551 S552 S558 Y574 S586 S590 T602 Y645 S655 Y668 S669 S705 T706 S713 S743 T759 S769 S774 S784 S789 S790 S791 S794 S796 S820 Y845 S869	S12 S15 S27 T28 T69 S71 S78 S82 T106 Y111 T118 T150 S153 S160 T169 S195 S196 S198 T230 S236 T239 T240 S243 S246 S251 T255 Y272 S274 S302 T319 T329 T354 S358 S362 T380 T404 S407 S409 S445 T485 T501 Y506 Y519 S527 T530 Y542 S551 S565 T570 S586 S590 S596 Y608 T635 Y645 S655 S662 Y668 S669 T693 T696 S703 S705 T706 T723 T725 T730 S743 S750 Y783 T846 Y849 T857 S869	S12 S15 S27 T28 T40 Y53 S67 T69 S82 T87 T88 S90 T92 T94 T97 T106 Y111 T114 S115 T118 T181 S184 S193 S195 Y228 S246 S251 T255 Y272 S274 S282 T306 T329 T380 S407 S445 Y462 T569 S590 S596 Y606 Y608 T635 S655 Y668 S703 S705 T706 S713 S721 T723 T725 T730 S743 S750 S774 Y783 S784 S789 S836 T857 T859
Channels	T895 Y896 S913 S921 S927 T929 S934 Y965 Y989 Y1023 S1031 Y1051 S1060 T1064 T1070 T1073 Y1080 Y1085	T895 Y896 S913 S921 S927 S929 Y949 Y953 Y989 Y1002 S1013 Y1023 S1029 S1031 Y1041 Y1051 S1060 T1064 Y1080 Y1085	S876 T881 S893 T913 T915 Y942 T974 S990 S1000 Y1022 S1034 S1035 T1046 Y1058	S893 S907 Y909 T913 T915 S922 Y942 S990 S1000 S1006 S1035 S1038 S1043 T1046 Y1053 Y1058
TRP	Y1100, S1107, Y016, Y1122, S1036, S1040, S1141	Y1100 S1107 Y016 Y1122 S1036 S1040 S1141	Y1073 Y1086 Y1089 Y1091 T1094 Y1095	Y1073 Y1086 Y1089 Y1091 T1094 Y1095 S1109
C-terminus	T1154 S1155 T1163 Y1181 S1191 S1255 S1258 T1265 S1269 S1271 S1292 T1296 S1298 S1299 S1300 S1308 S1349 S1350 S1360 S1587 S1888 S1590 Y1826 T1827 S1839 S1848 T1849 T1855	T1154 S1155 T1163 Y1181 S1191 S1193 S1255 **S1252** T1265 S1269 S1271 S1292 T1296 S1298 S1299 S1300 S1308 S1349 S1350 S1357 S1360 T1580 S1587 S1588 S1590 Y1826 T1827 S1839 S1848 T1849 S1852 T1855 S1857	S1168 T1229 S1244 T1245 T1304 S1306 T1311 T1322 S1324 S1325 S1339 S1365 S1368 T1375 S1395 S1399 T1426 S1970 T1983 Y1985 S1986 S2015	Y1137 S1139 S1226 T1230 S1244 T1245 Y1273 T1304 S1306 S1339 S1349 T1374 T1381 T1388 T1391 S1395 S1399 S1426 S1970 S1985 S1992 T1993 S2015
Coiled coil	T1200 S1208 S1224 S1227 T1242 T1245 T1248 T1250	T1200 Y1220 S1224 S1227 S1230 S1239 T1242 T1250	T1176 S1206 T1221 S1225	S1215 T1218 T1221
S/T rich domain	S1386 S1389 S1390 S1394 S1395 S1403 T1404 S1406 S1409 S1412 S1416 T1418 S1445 S1455 S1463 T1466 T1470 Y1479 T1485 T1487 S1488 S1491 T1493 S1495 S1497 S1501 T1502 S1505 S1510 T1524 S1530 T1534 S1540 T1548	S1386 S1389 S1390 S1394 S1395 S1403 T1404 S1406 S1409 S1412 S1416 T1418 Y1426 S1445 T1454 S1455 S1463 T1466 T1470 Y1479 T1485 T1487 S1488 S1491 T1493 S1495 S1497 S1501 T1502 S1505 S1510 T1524 S1530 T1534 S1540 T1548	S1428 T1430 S1437 S1438 S1441 T1448 S1458 S1503 T1504 S1506 S1513 T1523 S1524 Y1533 S1541 S1562 S1563 T1577 S1583 S1616 Y1622 S1623 T1628 S1630 S1685 S1689 S1690 S1697 S1699 Y1710 T1739 S1746 S1747	Y1452 T1463 S1467 T1474 S1478 T1479 S1482 S1484 S1485 S1487 S1497 T1504 S1506 S1510 S1513 T1523 S1524 S1539 S1560 S1563 T1577 S1583 T1589 T1598 S1603 Y1622 T1647 S1672 S1676 S1689 S1690 S1697 S1699 Y1741
Dimerization motif	S1553 S1564 S1566	S1553 S1564 S1566	S1722 T1724 T1728	Y1710 S1711
α -Kinase domain	S1595 S1597 S1598 S1600 S1612 T1629 S1631 Y1642 S1656 S1657 Y1659 T1663 S1692 Y1695 Y1696 S1709 Y1727 T1738 T1740 S1749 T1756 S1776 S1785	S1592 S1597 S1598 S1600 S1612 T1629 S1638 S1646 S1656 Y1659 T1663 T1682 S1692 Y1695 Y1696 S1709 Y1727 T1738 T1740 S1749 T1752 Y1753 Y1755 T1756 S1776 S1782 S1811	S1754 S1756 S1757 S1759 S1787 T1788 S1790 T1828 Y1842 **T1851** Y1854 T1855 Y1865 Y1878 T1897 T1899	S1754 S1756 S1757 S1805 S1821 T1822 Y1842 T1843 **T1851** T1880 T1895 T1911 Y1914 T1915 S1935 S1944

SP; spheroid, SP + low-Mg; SP in moderate low Mg^2+^ (0.4 mM), SP + high-Mg; SP in moderate high Mg^2+^ (2.5 mM), S; serine residue, T; threonine residue, Y; tyrosine residue.

**Table 2:** Phosphorylated residues of TRPM7 protein.

	Parental	SP	SP + Low-Mg	SP + High-Mg
N-terminus	S5 Y18 S22 Y62 S101 S103 Y108 S112 Y113 **S138** T166 T173 S196 Y225 S233 S243 Y256 T299 S307 T318 Y327 T332 S348 T349 T367 T379 S385 T403 S406 T414 Y430 S438 T485 Y524 T527 S539 S564 T583 Y587 T593 T675 S676 S683 S697 T710 S717 S719 T720 S727 S728 T737 T739 Y759 T795 S799 T807 S823 S836 T842 Y846 Y849	S5 T10 T12 Y18 S22 S23 T55 S63 S79 T84 T89 Y92 Y108 S112 Y113 T115 **S138** T166 T167 T173 T178 S196 T201 Y225 T227 S233 S243 Y256 T269 S307 T318 T397 S406 Y430 S438 Y478 T480 T485 Y528 T529 Y537 S539 T593 T603 T615 Y620 Y659 S671 S679 Y682 T710 S717 S719 T720 S757 S764 Y776 T778 S799 S836 T842 Y846 Y849	S2 Y18 S23 Y62 S63 T84 S112 Y113 T115 T173 T178 S196 Y225 T227 S243 T252 S307 T318 T353 T379 S385 S438 S464 T470 Y478 Y505 T508 Y518 T523 Y524 T527 Y528 T529 Y537 S539 S553 S554 T555 T603 T615 Y664 S683 S697 S717 S719 Y759 S788 S823 S836 Y849 Y863	S2 S5 T10 S22 S23 T55 S57 S63 S79 Y92 S112 S138 T166 T167 T173 T178 T201 S233 S243 T252 Y256 T299 Y303 S307 T318 T332 S348 T349 S438 S464 T485 T508 T523 Y524 T527 T529 S539 S547 T551 S552 S553 S554 T593 T603 T615 Y620 T675 S676 S683 S697 Y711 T737 T739 S744 S757 Y759 S764 Y776 T778 S783 S788 S827 S840
Channels	T895 S934 Y965 Y989 Y1002 S1013 S1029 T1031 Y1049 T1070 T1073 Y1080	Y896 S927 T929 S934 Y953 Y1002 S1029 T1031 Y1049 Y1051 S1060 T1070 T1073 Y1080	S883 S907 S921 S934 Y953 Y1002 S1013 Y1023 S1060 T1070 T1073 Y1080 Y1085	S883 Y892 T895 S921 S927 T929 S934 S1060 Y1080 Y1085 Y1089
TRP	Y1100 S1107 Y016 Y1122 S1036 S1040 S1141	Y1100 S1107 Y1113 Y1116 Y1122 S1136	S1140	Y1100 Y1116
C-terminus	S1191 S1193 S1258 T1265 S1269 S1271 S1300 S1309 S1351 S1352 S1358 **S1360** S1361 T1830 T1852 S1855	S1155 T1163 Y1181 S1255 S1258 T1265 S1269 S1300 S1301 S1309 S1352 **S1360** S1358 S1361 S1855 S1857 T1858	S1255 S1258 T1265 S1269 S1271 S1299 S1300 S1361 T1830 S1855 S1857 S1860	T1154 T1163 Y1181 S1191 S1193 S1255 S1258 S1301 S1351 S1352 S1358 S1361 T1830 S1842 S1851 S1857 T1858
Coiled coil	T1200 S1227 S1230 S1239 T1242	S1224 T1248 T1250	S1239 T1242	T1200 T1248 T1250
S/T rich domain	T1424 S1427 T1430 S1468 T1471 S1476 S1477 T1482 T1505 T1508 T1583 Y1585 S1590	S1390 S1395 S1396 T1399 S1404 T1405 S1407 S1410 S1413 T1417 T1419 T1430 T1435 S1446 S1476 S1477 T1505 T1508 S1513 T1583 S1590	S1413 T1417 T1419 T1435 S1446 T1482 T1487 S1500 S1504 T1537 Y1585	S1386 S1387 S1388 T1389 S1404 T1405 S1407 S1410 S1413 T1417 T1419 S1456 S1476 S1477 T1482 T1487 S1488 S1527 T1583 Y1585
Dimerization motif	T1551 Y1555 S1556	T1551 Y1554 Y1555 S1556 S1567	Y1554 Y1555	Y1555 S1569 T1573
α-Kinase domain	S1591 S1600 S1601 S1634 S1641 Y1645 S1649 T1657 S1659 Y1698 Y1709 S1712 Y1730 S1752 T1755 Y1758 T1759 T1776 S1779 S1788 S1814	S1591 S1600 S1601 T1632 S1634 S1641 Y1645 S1649 T1657 S1659 S1660 Y1662 T1666 T1685 Y1698 Y1709 S1712 Y1730 S1752 T1755 Y1758 T1776 S1779 S1814	S1600 S1601 S1603 S1641 Y1645 S1660 Y1662 T1666 T1685 S1695 T1755 Y1756 Y1758 S1779 S1788	S1600 S1601 S1603 S1615 S1649 T1657 S1659 S1660 Y1662 Y1698 S1699 Y1709 S1712 T1724 Y1730 T1741 T1743

SP; spheroid, SP + low-Mg; SP in moderate low Mg^2+^ (0.4 mM), SP + high-Mg; SP in moderate high Mg^2+^ (2.5 mM), S; serine residue, T; threonine residue, Y; tyrosine residue.

**Table 3:** Methionine oxidation in TRPM6 and TRPM7 proteins.

	Parental	SP	SP + Low-Mg	SP + High-Mg
TRPM6	M33 M63 M133 M338 M370 M618 M623 M625 M648 M657 M732 M768 M847 M864 M969 M984 M1020 M1061 M1076 M1093 M1162 M1278 M1183 M1434 M1436 M1575 M1879 M2020	M1 M133 M263 M338 M416 M618 M623 M625 M648 M657 M690 M692 M727 M732 M734 M739 M768 M780 M847 M854 M864 M969 M973 M977 M984 M1061 M1076 M1093 M1162 M1265 M1183 M1190 M1446 M1575 M1645 M1719 M1766 M1879 M1947	M1 M33 M133 M151 M244 M263 M338 M350 M370 M416 M450 M623 M625 M657 M690 M692 M727 M739 M847 M854 M969 M973 M977 M984 M1061 M1093 M1162 M1265 M1278 M1183 M1190 M1434 M1436 M1446 M1551 M1575 M1645 M1719 **M1755** M1766 M1775 M1947 M2020	M127 M151 M244 M338 M350 M450 M690 M692 M727 M732 M768 M847 M864 M969 M984 M1020 M1076 M1093 M1265 M2020 M1183 M1446 M1575 M1719 **M1755** M1766 M1783 M1904 M1947
TRPM7	M43 M60 M143 M372 M443 M449 M520 M595 M632 M649 M662 M704 M706 M741 M782 M812 M868 M878 M992 M1000 M1007 M1207 M1447 M1549 M1564 M1599 M1691 M1748 M1791	M1 M60 M130 M143 M369 M443 M449 M595 M632 M649 M662 M704 M706 M748 M753 M812 M830 M834 M992 M996 M1000 M1007 M1038 M1180 M1319 M1549 M1599 M1619 M1723 M1748 M1791	M1 M60 M130 M143 M369 M443 M449 M488 M520 M575 M595 M632 M662 M704 M706 M741 M746 M748 M753 M782 M796 M812 M868 M978 M906 M992 M996 M1000 M1007 M1043 M1088 M1120 M1180 M1447 M1531 M1549 M1564 M1599 M1619 M1691 M1723 M1748 M1791 M1864	M60 M143 M369 M372 M443 M465 M488 M575 M595 M632 M637 M746 M798 M800 M816 M834 M868 M906 M996 M1000 M1007 M1088 M1120 M1180 M1207 M1447 M1531 M1549 M1564 M1599 M1619 M1691 M1723 M1748 M1791

SP; spheroid, SP + low-Mg; SP in moderate low Mg^2+^ (0.4 mM), SP + high-Mg; SP in moderate high Mg^2+^ (2.5 mM), M; methionine residue.

## Data Availability

The data could be downloaded from the public databases, and no additional data are available. Further inquiries can be directed to the corresponding author.

## Abbreviations

The following abbreviations are used in this manuscript:

Bax	Bcl-2-associated X protein
Bcl-2	B-cell lymphoma 2
CRC	colorectal cancer
CNNM4	Cyclin and CBS Domain Divalent Metal Cation Transport Mediator 4
DXR	doxorubicin
MagT1	Magnesium Transporter 1
Mrs2	Mitochondrial inner-membrane magnesium transporter 2
ΔΨm	mitochondrial membrane potential
p53	tumor protein p53
p62/SQSTM1	Sequestosome-1
S	serine residue
SP	spheroid
T	threonine residue
TME	tumor microenvironment
TRPM	Transient Receptor Potential Melastatin
ULK1	Unc-51-like kinase 1
Y	tyrosine residue
